# Supplementary material for: Pan-cancer characterization of immune-related lncRNAs identifies potential oncogenic biomarkers
Source: Nat Commun. 2020 Feb 21;11:1000. doi: 10.1038/s41467-020-14802-2 (PMC7035327; doi:10.1038/s41467-020-14802-2)
Supplement: Supplementary file 2 — Supplementary Information [file 41467_2020_14802_MOESM2_ESM.pdf]

Pan-cancer characterization of immune-related lncRNAs identifies potential oncogenic biomarkers

Li., *et al*

Supplementary Methods

Independent Datasets

We obtained several independent datasets from other public resources. We downloaded the raw RNA-Seq data and obtained the lncRNA and gene expression profiles for LGG and GBM in Chinese Glioma Genome Atlas (CGGA) projects[1]. The detailed processes have been described in one of our previous studies[2]. The lncRNA and gene expression profiles in LIHC, OV, and PRAD were downloaded from the International Cancer Genome Consortium (ICGC)[3], and data for LUAD were obtained from GEO (Accession number: GSE40419)[4].

Moreover, we curated lncRNA and mRNA expression levels in different immune cell populations. First, we obtained the expression across 63 samples from one recent study[5]. Moreover, we obtained the expression from the Database of Immune Cell Expression (DICE), expression quantitative trait loci (eQTLs) and Epigenomics[6]. Based on these datasets of immune cell populations, we identified the lncRNA–pathway associations and analyzed overlap with those identified in TCGA. The hypergeometric test was used to evaluate the significance.

Estimate Tumor Purity

Estimation of STromal and Immune cells in MAlignant Tumor tissues using Expression data (ESTIMATE) was used to estimate the immune score, stromal score, and tumor purity of each patient across 33 cancer types[7].

Identification of lncRNAs with Expression Perturbation in Cancer

We used two methods to identify differentially expressed lncRNAs in each cancer type[8]. Here, we only considered the 17 cancer types with more than five normal samples. LncRNAs with zero expression in less than 30% of the samples were subjected to a Student's *t*-test. LncRNAs with zero expression in

more than 30% of the samples were used for on/off analysis. For each lncRNA, we determined in a binary fashion: ON (expressed, FPKM > 0), OFF (not expressed, FPKM = 0). Fisher's exact test was used to evaluate whether the distribution of samples was different. LncRNAs with false discovery rate (FDR) less than 0.01 were identified as differentially expressed lncRNAs. In order to evaluate whether the expression of immune-related lncRNAs was likely to be perturbed, we compared the proportion of differentially expressed lncRNAs with all lncRNAs using Fisher's exact test. The odds ratios (ORs) and 95% confidence levels were also calculated.

Cancer Similarity Score based on Immune-related lncRNAs

To evaluate the similarity of cancer types based on the immune-related lncRNA regulators, we calculated the Jaccard index for each pair of cancer as follow:

$$Jaccard\ index\ (C_i, C_j) = \frac{ImmLnc_i \cap ImmLnc_j}{ImmLnc_i \cup ImmLnc_j}$$

where $ImmLnc_i$ and $ImmLnc_j$ are the immune-related lncRNA regulators in cancer types *i* and *j*, respectively. We clustered the cancer similarity matrix and viewed the results using the R package ape (Analyses of Phylogenetics and Evolution)[9].

Identification of Immune Cell Infiltration-related lncRNAs

Levels of six tumor-infiltrating immune subsets (i.e., B cells, CD8 T cells, CD4 T cells, macrophages, neutrophils, and dendritic cells) were estimated by Tumor Immune Estimation Resource (TIMER)[10, 11]. To identify the immune cell infiltration-related lncRNAs, we calculated the Spearman correlation coefficient (SCC) between the expression of lncRNA and the immune cell proportion in cancer patients. The lncRNAs with an absolute value of SCC > 0.3 and $P < 0.05$ were considered as immune cell infiltration-related lncRNAs. We first

calculated the proportion of lncRNAs, which was defined as the number of immune-related lncRNAs that were correlated with immune cell infiltration level divided by the total number of immune cell infiltration-related lncRNAs. Next, we used Fisher's exact test to evaluate whether the lncRNA immune regulators were enriched in immune cell infiltration-related lncRNAs.

In addition, we estimated the immune cell proportion in each patient based on CIBERSORT[12]. We identified the lncRNAs whose expression was significantly correlated with immune cell infiltration in each cancer type. The hypergeometric test was used to evaluate the overlap between identified lncRNAs based on TIMER and CIBERSORT.

Validation of the ImmLnc Pipeline

As the number of validated immune-related lncRNAs is limited, we validated the pipeline of ImmLnc indirectly. With the development of high-throughput sequencing data, in particular CRISPR interference, several lncRNAs essential for cell growth have been identified. We hypothesized that if this pipeline can accurately identify the lncRNAs that are essential for cell growth, we can extend it to immunology-related functions. The essential lncRNAs in four cancer cell lines (K562, U87, MCF7 and MDA-MB-231) were obtained by large-scale CRISPR screening[13] and the cell growth-related gene set was downloaded from MSigDB[14]. Next, we applied the ImmLnc pipeline to identify lncRNAs that were likely to regulate the cell growth. All lncRNAs were ranked based on the *P*-values and the lncRNA ranks were normalized. The difference in relative rank for essential lncRNAs and other lncRNAs was compared by the one-side Wilcoxon Rank-Sum test in BRCA, GBM, LGG and LAML.

In addition, recent studies have suggested that genes, whose expression is negatively correlated with tumor purity and positively correlated with immune cell infiltration, are likely to play crucial roles in immunology[11, 15]. Therefore, we identified these lncRNAs in each cancer type. We next evaluated whether the

lncRNAs were enriched those lncRNAs. Fisher's exact test was used for this procedure and the ORs and *P*-values were calculated.

Expression of lncRNAs in Immune Cells

To investigate the expression of lncRNAs in immune cell populations, we first downloaded the expression of single cells from one recent study[16]. We calculated the average expression of each lncRNA as identified by the authors in B cells and T-cells. We used the Wilcoxon Rank-Sum test to evaluate the difference between the expression of immune-related and other lncRNAs. In addition, to evaluate whether the lncRNAs used for pan-lung cancer classification exhibit higher expression in immune cells, we calculated the average expression of these lncRNAs in B cells and T cells. Next, we randomly selected the same number of lncRNAs from the total lncRNAs. The average expression levels were recalculated and this procedure was repeated 100,000 times. We defined the *P*-values as follows:

$$P = \frac{\#(E_i > E_r)}{100,000} \quad ,i=1,2,3,...100000.$$

where E_r is the average expression of lncRNAs and E_i is the average expression in random conditions.

Moreover, we downloaded 10 datasets based on 10X Genomics Chromium from PanglaoDB[17], which is a database for collecting single-cell sequencing studies. Only approximately 10% of lncRNAs showed expression in these datasets, consistent with one recent study[18]. We used Fisher's exact test to evaluate whether the immune-related lncRNAs identified by ImmLnc were likely to be expressed in immune cells. We first calculated the average expression of lncRNAs across immune cells; if the average expression of a lncRNA > 0, we defined that this lncRNA was expressed in immune cells. Next, a contingency table was constructed as follows:

	Immune lncRNAs	Nonimmune lncRNAs
Expressed	a	c
Not expressed	b	d

The OR, 95% confidence level of the OR and *P*-values were calculated for each cancer type.

Reconstruction of lncRNA Expression in Immune Cell Types

Moreover, we reconstructed the immune cell-specific expression of lncRNAs from bulk RNA-Seq data based on the ideas of RESPECTEx[15]. The lncRNA expression in tumor patients was deconvoluted by means of a linear regression. We hypothesized that in each patient, each cell type present contributes a variable level of lncRNA expression to the observed value. The contribution of each immune cell type was weighted by the proportion of the cell type present in the tumor patient. This can be represented mathematically as follows:

$$\boldsymbol{B}_{g,s} = \left[\boldsymbol{\beta}_{g,1}, \boldsymbol{\beta}_{g,2}, \boldsymbol{\beta}_{g,3}, \dots, \boldsymbol{\beta}_{g,c}\right] \begin{bmatrix} \boldsymbol{x}_{1,s} \\ \boldsymbol{x}_{2,s} \\ \boldsymbol{x}_{3,s} \\ \dots \\ \boldsymbol{x}_{c,s} \end{bmatrix}$$

where $\boldsymbol{B}_{g,s}$ is the observed lncRNA expression value in the bulk RNA-Seq data for gene *g* in sample *s*, $\boldsymbol{\beta}_{g,1}, \boldsymbol{\beta}_{g,2}, \boldsymbol{\beta}_{g,3}, \dots, \boldsymbol{\beta}_{g,c}$ are the mean expression values for gene *g* in the cell types, and $\boldsymbol{x}_{1,s}, \boldsymbol{x}_{2,s}, \boldsymbol{x}_{3,s}, \dots, \boldsymbol{\beta}_{c,s}$ are the proportions of all cell types in sample *s*. The tumor cell proportion was defined as the tumor purity. The immune cell subpopulations were inferred by TIMER[10], which was adjusted by multiplying (1-tumor purity).

For each cancer type, we calculated the ratio between the average expression of immune-related lncRNAs and other lncRNAs. The difference was evaluated by Wilcoxon Rank-Sum test. In addition, we performed the

same analysis for lncRNAs that were correlated with immune cell infiltration in different cancer types.

Tissue Specificity of lncRNA Expression

Based on the expression of lncRNAs across different cancer types, all the human lncRNAs were classified into five classes based on the method that had been applied to coding gene[19, 20]. (i) Tissue-specific (TS) lncRNAs, which are expressed only in a particular cancer type; (ii) tissue-enriched (TER) lncRNAs, which show at least fivefold higher expression in a particular cancer type compared to other cancers; (iii) group enriched (GER) lncRNAs, which show at least fivefold higher expression in a group of cancer types (n = 2–7); (iv) tissue enhanced (TEH) lncRNAs, which show at least fivefold higher expression in a particular cancer type compared to the average expression level in all cancers; and (v) other lncRNAs. The lncRNAs in group i–iv were defined as tissue elevated (TE) lncRNAs. Next, we evaluate whether the immune-related lncRNAs were significantly overlapped with the TE lncRNAs in each cancer type by hypergeometric test to. The observed/expected values (O/E) and *P*-values were calculated.

Literature Curation of lncRNAs

To investigate whether the identified lncRNAs were correlated with immunology or cytokines, we queried PubMed to check whether they co-occurred with “immune” or “cytokine” in the literature. This process was performed using the R package ‘RISmed’ (https://rdrr.io/cran/RISmed/). We calculated the proportion of lncRNAs that were co-occurred with “immune” or “cytokine” of immune-related lncRNAs and other lncRNAs. The differences were evaluated by Fisher’s exact test. OR and *P*-values were calculated.

Immune-related Scores of Cancer Patients

We calculated three immune response-related scores that were estimated using gene expression. First, the immune score, which represents the infiltration of immune cells in tumor tissues, was estimated using the R package ESTIMATE[7]. Second, the MHC score was estimated using gene expression of the “core” MHC-I set (including *HLA-A*, *HLA-B*, *HLA-C*, *TAP1*, *TAP2*, *NLRC5*, *PSMB9*, *PSMB8*, and *B2M*) obtained from a recent study[21]. The FPKMs of these genes were first log-transformed and then median-centered. The mean expression of these core MHC-I genes was defined as the MHC score for patients. Similar as a previous study[22], we quantitatively measured of immune cytolytic activity (CYT) based on expression levels of granzyme A (*GZMA*) and perforin (*PRF1*).

In addition, we obtained another 160 immune expression signature scores from one recent study[23]. The difference of these immune-related scores between different cancer subtypes was evaluated by the one-way analysis of variance (ANOVA). We ranked these immune-related signatures by the *P*-values of ANOVA.

Construction of ImmLnc resource

The database was organized by MySQL (version 5.5.21) and queried using JavaServer Pages (JSP). The web interface was developed using HTML5 with JavaScript. All data in ImmLnc were stored and managed using MySQL (version 5.5.21). The web interface was built in JSP. The data processing programs were written in Java (version 1.7.0_80), and the web services were built using Apache Tomcat. The ImmLnc database is freely available at http://bio-bigdata.hrbmu.edu.cn/ImmLnc.

Supplementary Figures

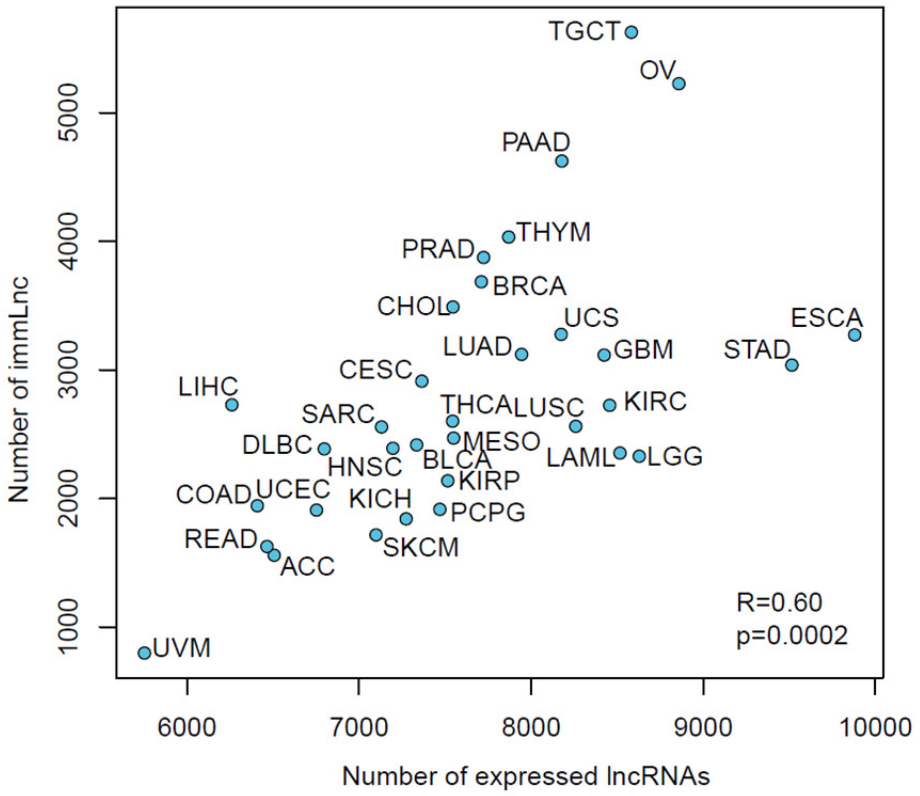

Supplementary Figure 1. The number of immune-related lncRNAs and expressed lncRNAs in different cancer types. The *x*-axis shows the number of expressed lncRNAs in each cancer type, and the *y*-axis shows the number of immune-related lncRNAs identified by the ImmLnc pipeline. Each dot represents one cancer type. The Pearson Correlation Coefficient (PCC) is 0.60 and *P* = 0.0002.

a

Immune pathways

Cancer types	1	2	3	4	5	6	7	8	9	10	11	12	13	14	15	16	17
BRCA	0.1182	0.0968	0.0144	0.0484	0.1989	0.0004	0.0085	0.0352	0.0000	0.0292	0.2100	0.0038	0.0637	0.0136	0.0226	0.0556	0.0552
CHOL	0.0929	0.1115	0.0062	0.0883	0.1662	0.0007	0.0066	0.0253	0.0012	0.0453	0.2432	0.0013	0.0197	0.0072	0.0013	0.0186	0.0150
LIHC	0.1341	0.1181	0.0088	0.0421	0.1489	0.0000	0.0035	0.0085	0.0029	0.0351	0.1395	0.0019	0.0099	0.0027	0.0034	0.0200	0.0610
PAAD	0.0906	0.3229	0.0237	0.1129	0.3181	0.0007	0.0159	0.0166	0.0117	0.0555	0.2841	0.0016	0.0321	0.0081	0.0407	0.0452	0.1015
BLCA	0.0881	0.1045	0.0067	0.0440	0.1769	0.0004	0.0223	0.0320	0.0019	0.0118	0.0968	0.0004	0.0396	0.0078	0.0030	0.0457	0.0464
KICH	0.0517	0.1086	0.0346	0.0440	0.0932	0.0000	0.0082	0.0056	0.0082	0.0397	0.0770	0.0029	0.0047	0.0037	0.0041	0.0525	0.0665
KIRC	0.0316	0.0709	0.0001	0.0287	0.1593	0.0000	0.0028	0.0281	0.0021	0.0130	0.1592	0.0021	0.0139	0.0101	0.0048	0.0106	0.0248
KIRP	0.0553	0.0395	0.0031	0.0242	0.1176	0.0013	0.0037	0.0074	0.0050	0.0367	0.1168	0.0005	0.0113	0.0133	0.0050	0.0148	0.0250
PRAD	0.0578	0.2151	0.0102	0.1439	0.2743	0.0003	0.0097	0.0162	0.0008	0.1643	0.3158	0.0068	0.0454	0.0124	0.0067	0.0286	0.0471
TGCT	0.1958	0.0797	0.0045	0.0288	0.3579	0.0007	0.0023	0.2971	0.0013	0.0116	0.2815	0.0016	0.0075	0.0070	0.0122	0.0250	0.0899
CESC	0.0908	0.1157	0.0079	0.0198	0.0956	0.0019	0.0184	0.0372	0.0024	0.0412	0.1867	0.0033	0.0327	0.0030	0.0065	0.0519	0.0540
OV	0.1423	0.1193	0.0167	0.0215	0.0828	0.0009	0.0096	0.0724	0.0053	0.1856	0.3909	0.0030	0.0421	0.0072	0.0037	0.0711	0.0475
UCEC	0.0608	0.0675	0.0021	0.0149	0.0796	0.0010	0.0053	0.0105	0.0024	0.0182	0.1593	0.0013	0.0099	0.0052	0.0047	0.0133	0.0217
UCS	0.0669	0.1516	0.0046	0.0499	0.1703	0.0011	0.0029	0.0394	0.0042	0.0153	0.1578	0.0031	0.0108	0.0223	0.0148	0.0412	0.0181
SKCM	0.0456	0.0917	0.0125	0.0371	0.1498	0.0010	0.0075	0.0294	0.0017	0.0183	0.0731	0.0034	0.0286	0.0035	0.0056	0.0302	0.0325
SARC	0.1186	0.0755	0.0031	0.0139	0.0886	0.0008	0.0064	0.0185	0.0014	0.0485	0.0957	0.0071	0.0048	0.0156	0.0094	0.0199	0.0378
DLBC	0.1264	0.0509	0.0025	0.0075	0.0696	0.0015	0.0046	0.0051	0.0049	0.0093	0.0884	0.0019	0.0143	0.0044	0.0140	0.0253	0.0794
LAML	0.0351	0.0250	0.0061	0.0230	0.0919	0.0028	0.0020	0.0073	0.0057	0.0039	0.1211	0.0155	0.0039	0.0083	0.0068	0.0109	0.0641
THYM	0.0591	0.0513	0.0128	0.0454	0.1837	0.0008	0.0038	0.0609	0.0028	0.0073	0.2287	0.0020	0.0077	0.0863	0.0260	0.0585	0.1710
GBM	0.0993	0.0673	0.0107	0.0157	0.1173	0.0000	0.0104	0.0037	0.0007	0.0704	0.1347	0.0024	0.0052	0.0037	0.0049	0.0172	0.0064
LGG	0.0668	0.0559	0.0253	0.0054	0.0919	0.0002	0.0037	0.0010	0.0031	0.0116	0.0866	0.0018	0.0035	0.0069	0.0025	0.0093	0.0189
UVM	0.0384	0.0276	0.0010	0.0144	0.0388	0.0000	0.0007	0.0094	0.0005	0.0127	0.0222	0.0024	0.0016	0.0030	0.0113	0.0193	0.0090
HNSC	0.0765	0.0803	0.0136	0.0083	0.1209	0.0008	0.0136	0.0437	0.0026	0.0305	0.1178	0.0008	0.0253	0.0046	0.0032	0.0312	0.0476
ACC	0.0709	0.0624	0.0012	0.0128	0.0735	0.0000	0.0026	0.0180	0.0020	0.0034	0.0355	0.0022	0.0043	0.0042	0.0012	0.0235	0.0337
PCPG	0.0653	0.0780	0.0021	0.0339	0.1224	0.0011	0.0025	0.0029	0.0054	0.0189	0.1044	0.0013	0.0119	0.0033	0.0035	0.0141	0.0166
THCA	0.0603	0.1527	0.0021	0.1165	0.1738	0.0005	0.0016	0.0091	0.0031	0.0729	0.2174	0.0012	0.0431	0.0039	0.0197	0.0575	0.0256
LUAD	0.1235	0.0571	0.0089	0.0119	0.0735	0.0005	0.0039	0.0133	0.0009	0.0377	0.2057	0.0019	0.0103	0.0096	0.0084	0.0185	0.0277
LUSC	0.0360	0.0751	0.0157	0.0176	0.1133	0.0001	0.0117	0.0197	0.0006	0.0455	0.1819	0.0167	0.0143	0.0031	0.0102	0.0222	0.0250
MESO	0.1164	0.0624	0.0105	0.0148	0.0670	0.0019	0.0050	0.0323	0.0024	0.0089	0.0652	0.0019	0.0167	0.0162	0.0093	0.0528	0.0742
ESCA	0.0352	0.0724	0.0100	0.0104	0.0722	0.0009	0.0169	0.0163	0.0025	0.0439	0.2196	0.0025	0.0282	0.0053	0.0088	0.0418	0.0456
STAD	0.0311	0.0427	0.0149	0.0151	0.0816	0.0013	0.0049	0.0311	0.0005	0.0162	0.2150	0.0008	0.0145	0.0059	0.0060	0.0216	0.0333
COAD	0.1137	0.0389	0.0047	0.0079	0.0860	0.0005	0.0036	0.0075	0.0017	0.0314	0.1158	0.0005	0.0101	0.0062	0.0212	0.0115	0.0132
READ	0.0421	0.0294	0.0028	0.0118	0.0431	0.0003	0.0043	0.0209	0.0022	0.0271	0.1163	0.0019	0.0113	0.0206	0.0306	0.0152	0.0203

Pathways: 1-Antigen Processing and Presentation
2-Antimicrobials
3-BCR Signaling Pathway
4-Chemokines
5-Cytokines
6-Interferons
7-Interleukins
8-TGFb Family Member
9-TNF Family Members
10-Chemokine Receptors
11-Cytokine Receptors
12-Interferon Receptor
13-Interleukins Receptor
14-TGFb Family Member Receptor
15-TNF Family Members Receptors
16-Natural Killer Cell Cytotoxicity
17-TCR Signaling Pathway

b

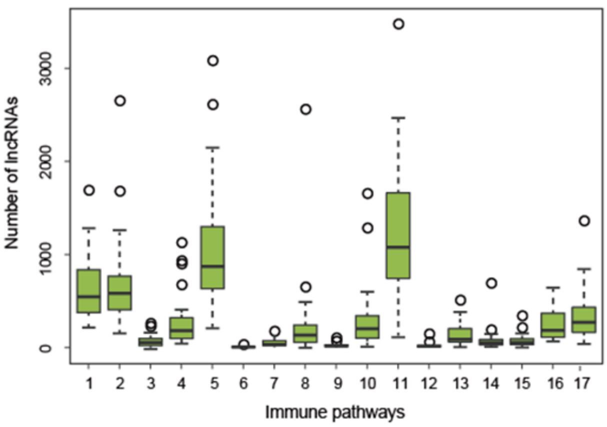

Supplementary Figure 2. The immune-related lncRNAs in different cancer types. a. Heat map showing the proportion of lncRNAs that are correlated with each immune pathway activity in different cancer types. Rows, cancer types; columns, immune-related pathways. b. Box plots showing the distribution of the number of lncRNAs correlated with in 33 cancer types. Numbers 1–17 represent immune pathways listed in the left panel. The Centre of the boxplots are median values, the bounds of the boxes are 25% and 75% quantiles. The minima are 25% quantile-1.5*interquartile range (IQR) and the maxima are 75% quantile+1.5*IQR.

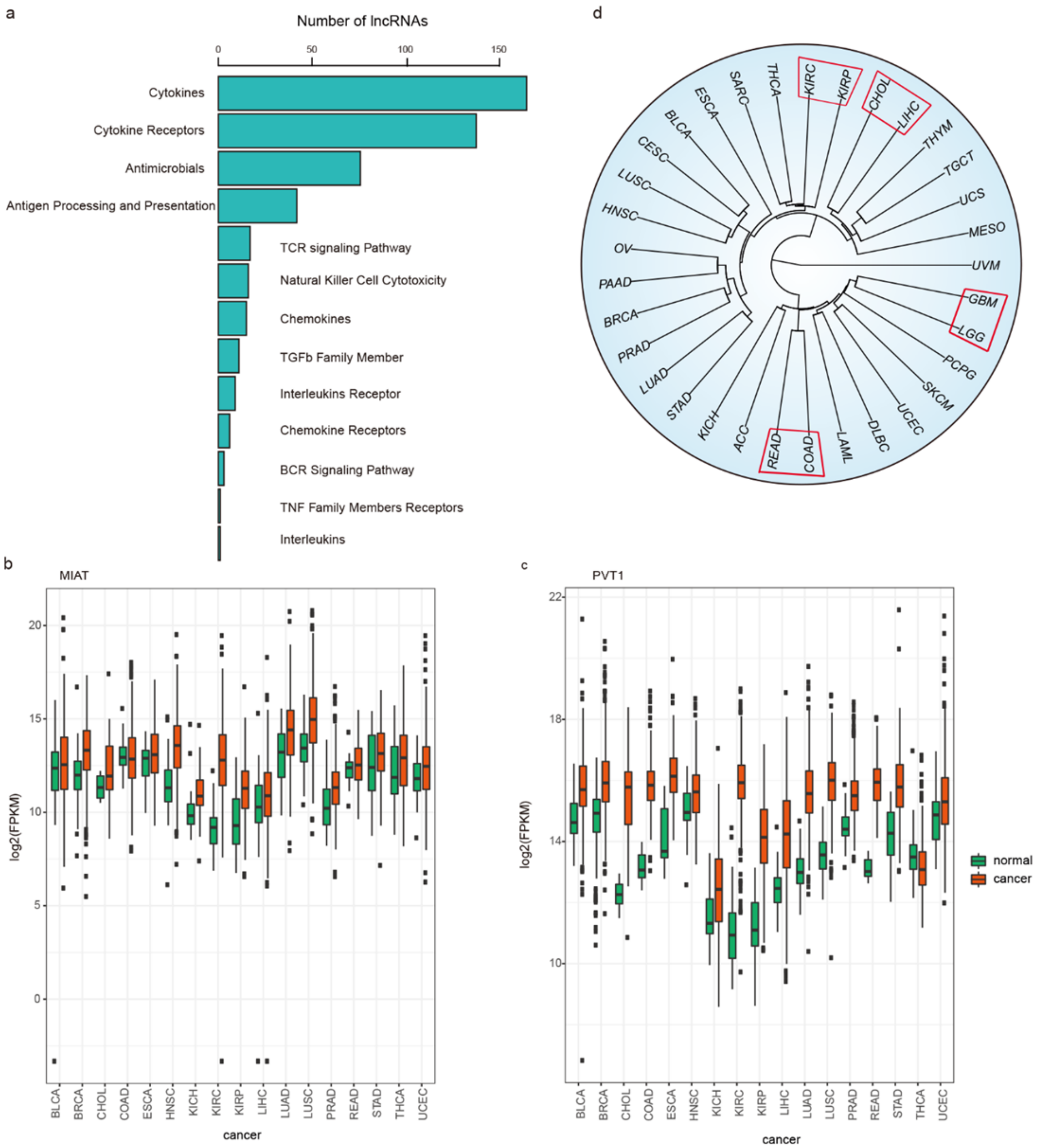

Supplementary Figure 3. The transcriptome perturbation of immune-related lncRNAs. a. Bar plot showing the number of lncRNAs correlated with each pathway in the top-ranked lncRNA–pathway pairs. b. Box plots showing the distribution of *MIAT* in normal and cancer samples. c. Box plots showing the distribution of *PVT1* in normal and cancer patients. d. Cluster of cancer types based on the proportion of shared immune-related lncRNAs. The Centre of the boxplots are median values, the bounds of the boxes are 25% and 75% quantiles. The minima are 25% quantile-1.5*interquartile range (IQR) and the maxima are 75% quantile+1.5*IQR.

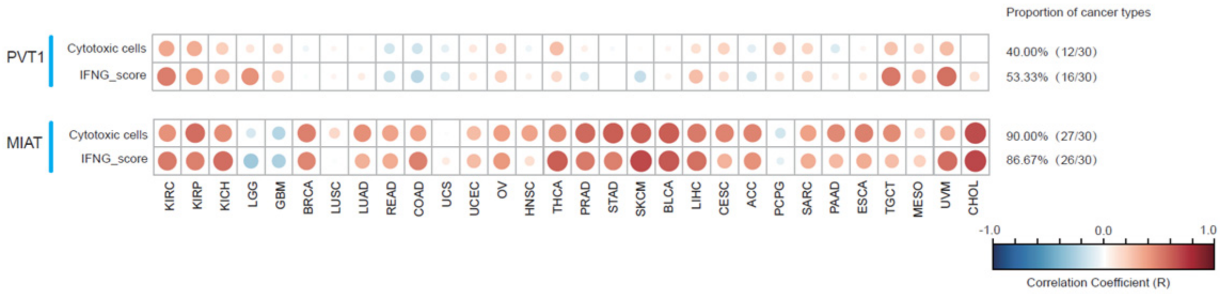

Supplementary Figure 4. The correlation between lncRNA expression and cytokine-related pathway activities in cancer.

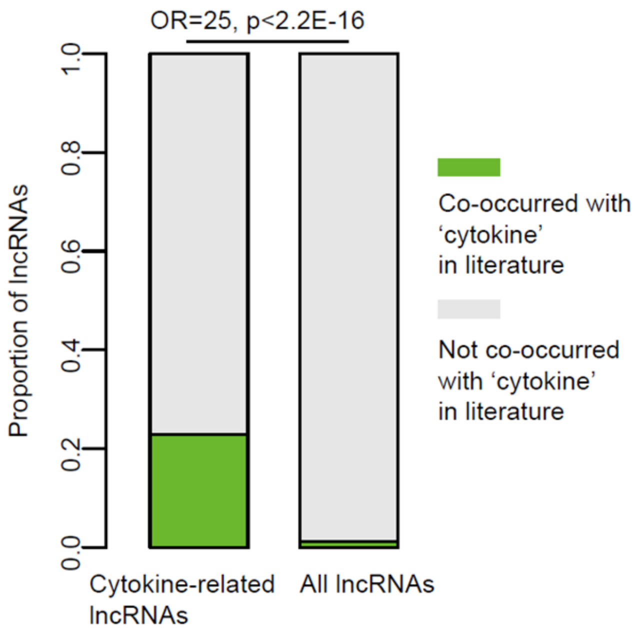

Supplementary Figure 5. The proportion of lncRNAs that co-occurred with "cytokine" in the literature. Two-sided Fisher's exact test was used to evaluate the difference.

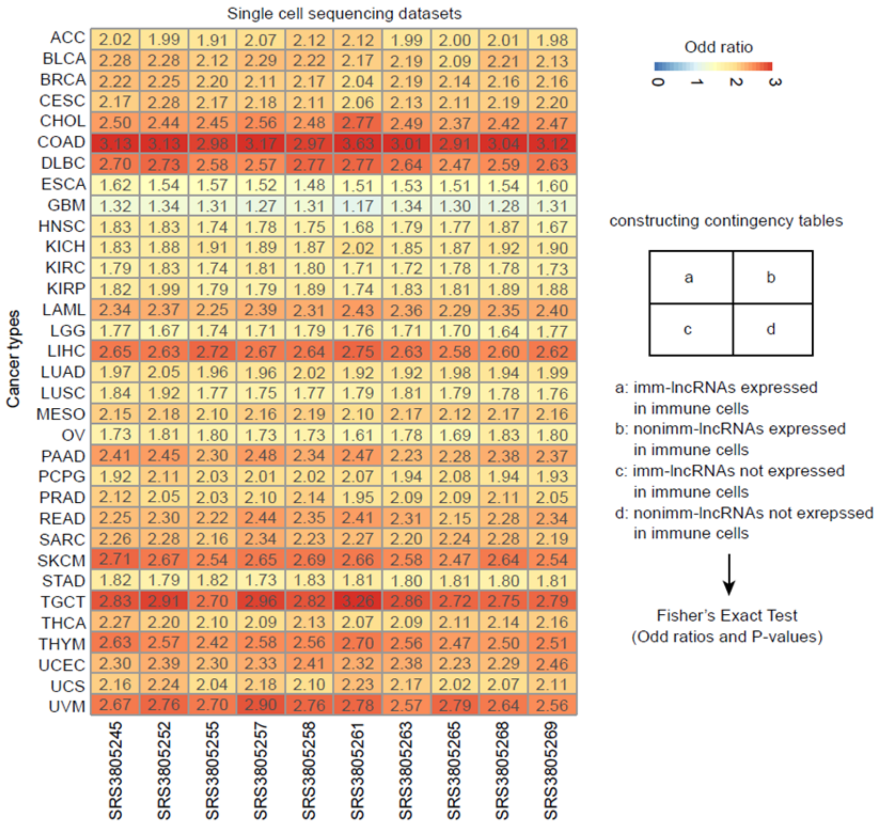

Supplementary Figure 6. Heat map showing odd ratios of Fisher's exact test. The proportions of immune lncRNAs and nonimmune lncRNAs expressed in immune cell populations were compared in 10 single-cell sequencing datasets. All $P < 0.001$, two-sided Fisher's exact test.

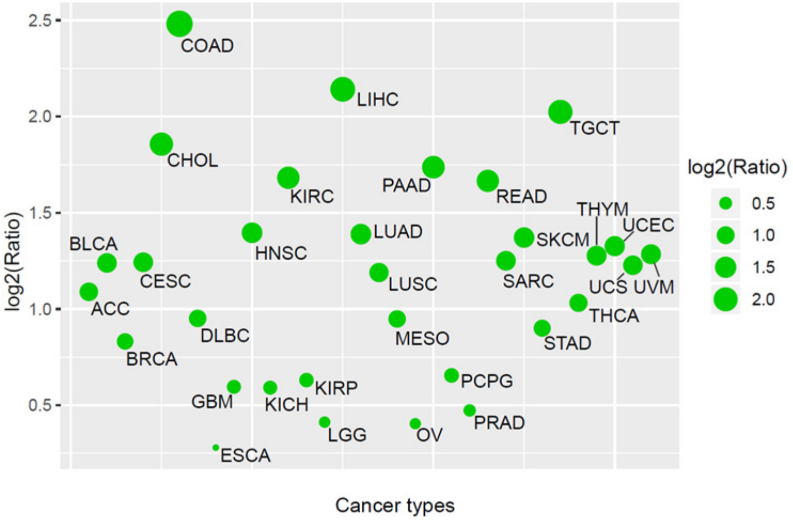

Supplementary Figure 7. The enrichment of immune-related lncRNAs expressed in immune cell populations. The *y*-axis shows the $\log_2$(ratio) between average expression of immune-related lncRNAs and other lncRNAs in immune cells. Green indicates the *P*-values for Wilcoxon Rank-Sum tests were less than 0.01.

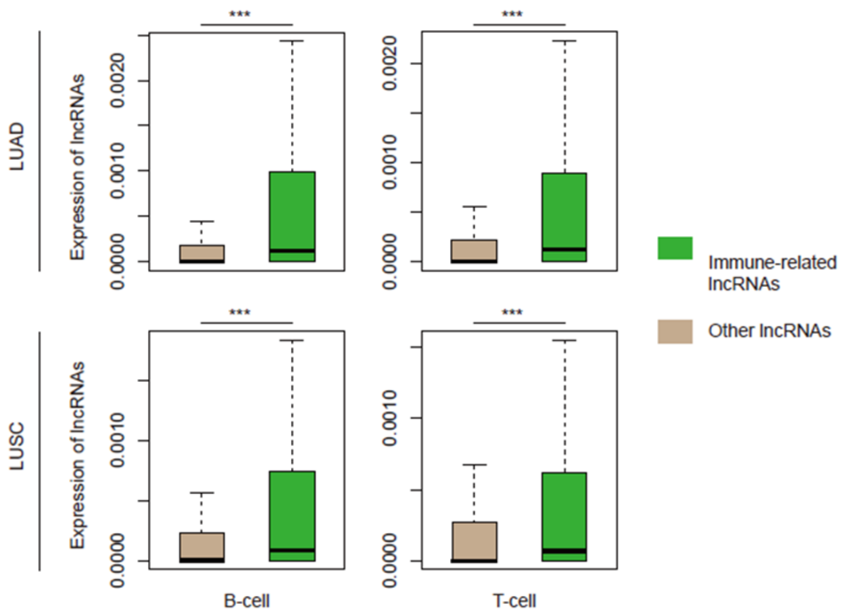

Supplementary Figure 8. The expression of immune-related lncRNAs in immune cell populations. The top panels show lncRNAs identified in LUAD and the bottom panels show lncRNAs identified in LUSC. ****P* < 2.2E-16, two-sided Wilcoxon Rank-Sum test. The Centre of the boxplots are median values, the bounds of the boxes are 25% and 75% quantiles. The minima are 25% quantile-1.5*interquartile range (IQR) and the maxima are 75% quantile+1.5*IQR.

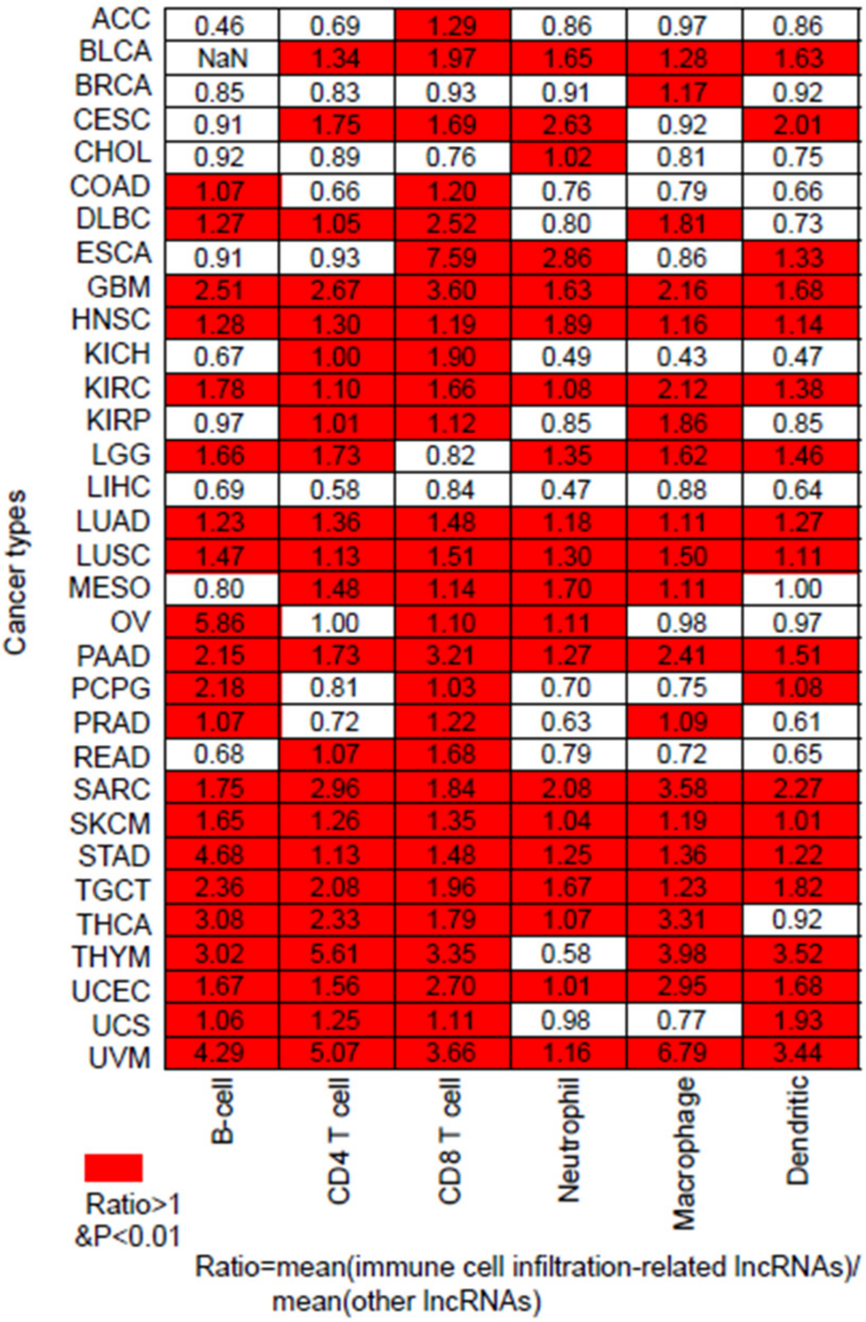

Supplementary Figure 9. Heat map showing the ratio between average expression of immune cell infiltration-related and other lncRNAs. Two-sided Wilcoxon Rank-Sum tests were used.

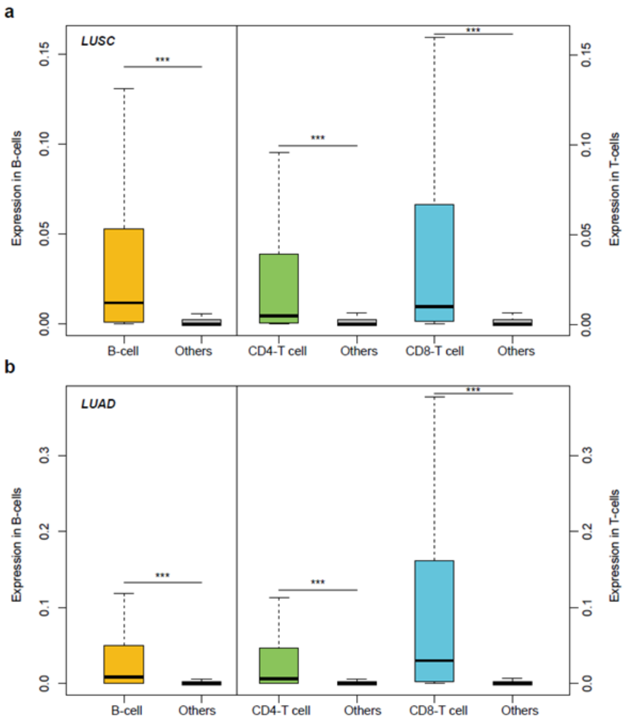

Supplementary Figure 10. The expression of lncRNAs in immune cell populations. a. The expression of lncRNAs that were correlated with immune cell infiltration in LUSC. b. The expression of lncRNAs that were correlated with immune cell infiltration in LUAD. ***P < 2.2E-16, two-sided Wilcoxon Rank-Sum tests. The Centre of the boxplots are median values, the bounds of the boxes are 25% and 75% quantiles. The minima are 25% quantile-1.5*interquartile range (IQR) and the maxima are 75% quantile+1.5*IQR.

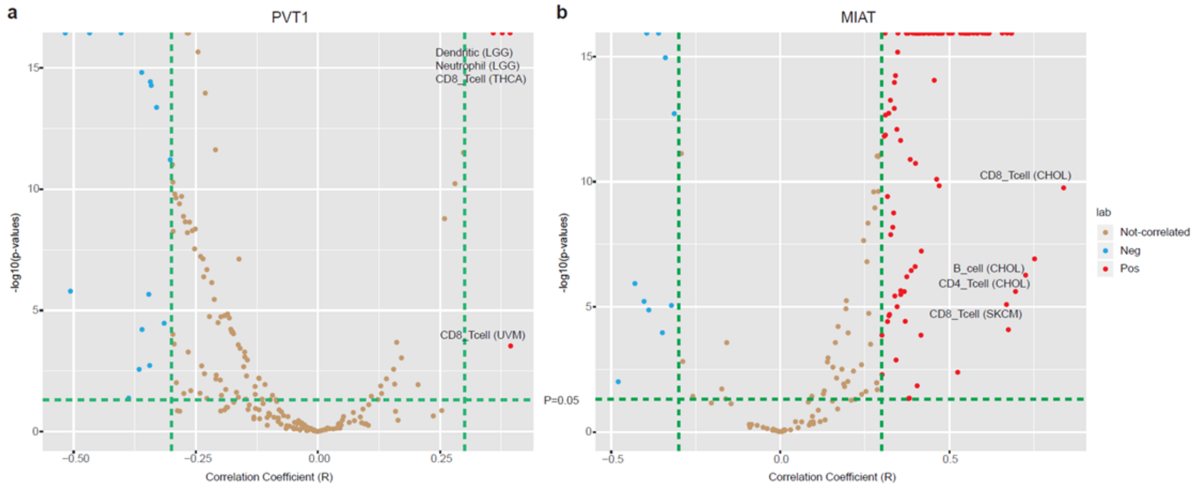

Supplementary Figure 11. The correlation between expression of lncRNAs and immune cell infiltration. The *x*-axis represents the Spearman Correlation Coefficient (SCC), and the *y*-axis represents the -log10(*P*-value). Each dot represents an immune cell type in one cancer. a, *PVT1*; b, *MIAT*.

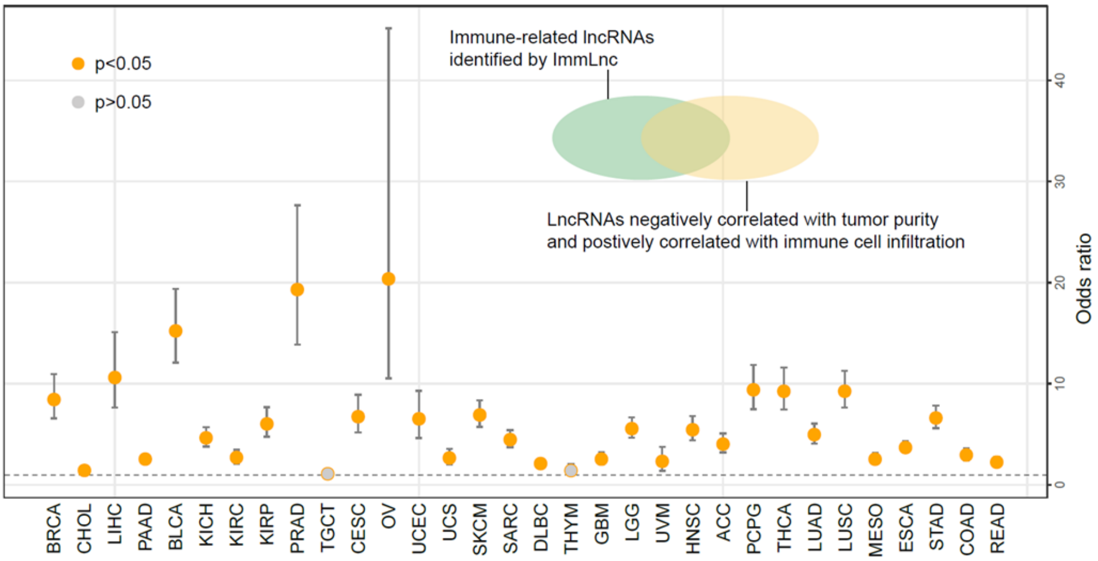

Supplementary Figure 12. The overlap of immune-related lncRNAs with lncRNAs negatively correlated with tumor purity and positively correlated with immune cell infiltration. The error bars were the 95% confidence level of the odds ratio. Two-sided Fisher's exact tests were used.

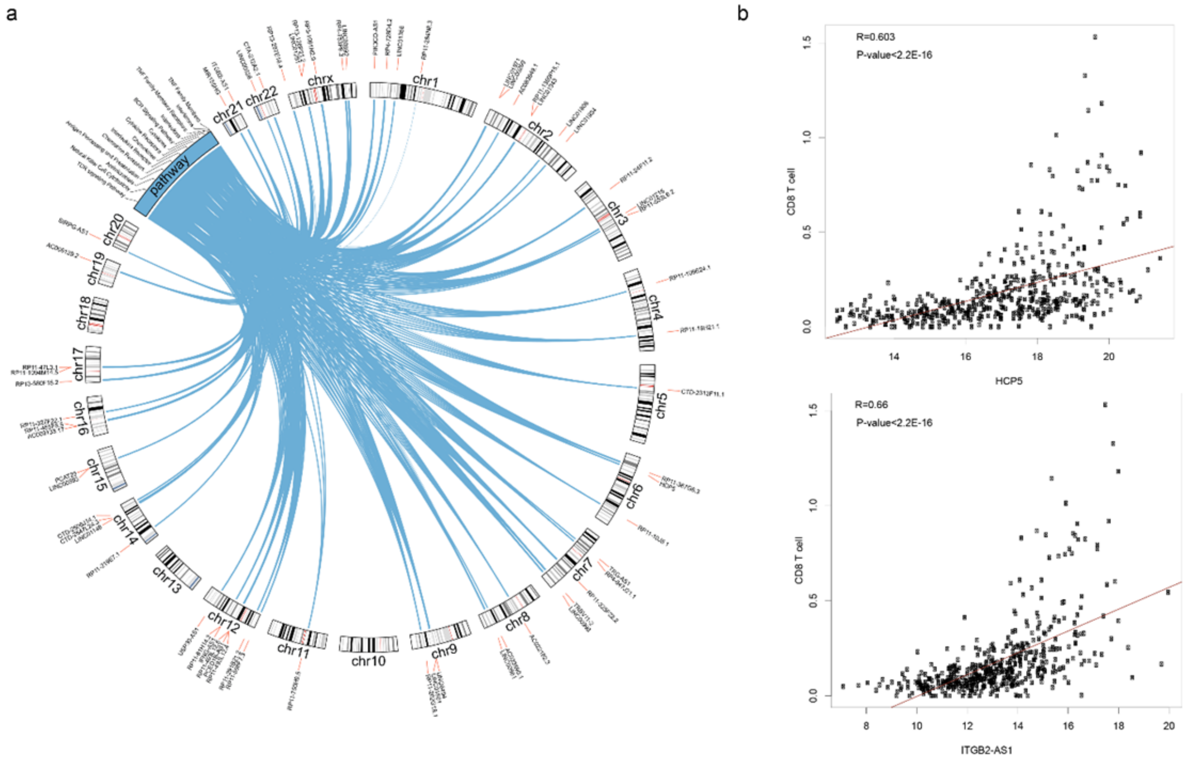

Supplementary Figure 13. The CD8 T cell-related lncRNAs in SKCM. a. Circos plot showing the correlation between lncRNAs and immune pathways. The CD8 T cell infiltration-related lncRNAs in SKCM are shown. b. Scatter plots showing the correlation between the expression of two lncRNAs and CD8 T cell infiltration in SKCM patients.

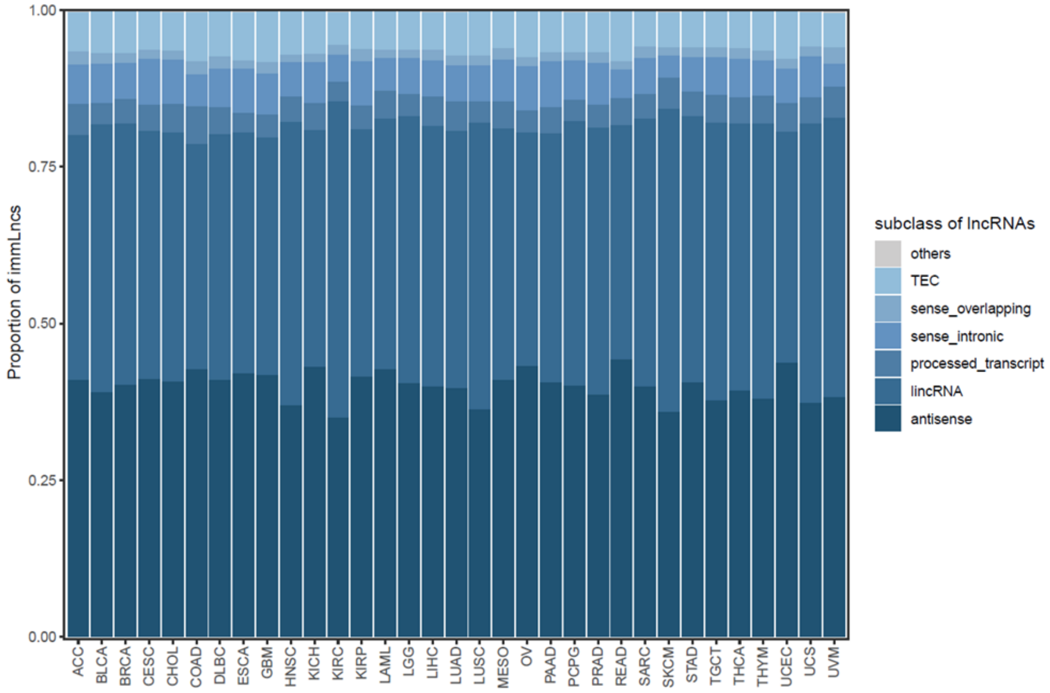

Supplementary Figure 14. The proportion of lncRNAs in different subtypes. The lncRNA classification information was obtained from GENCODE.

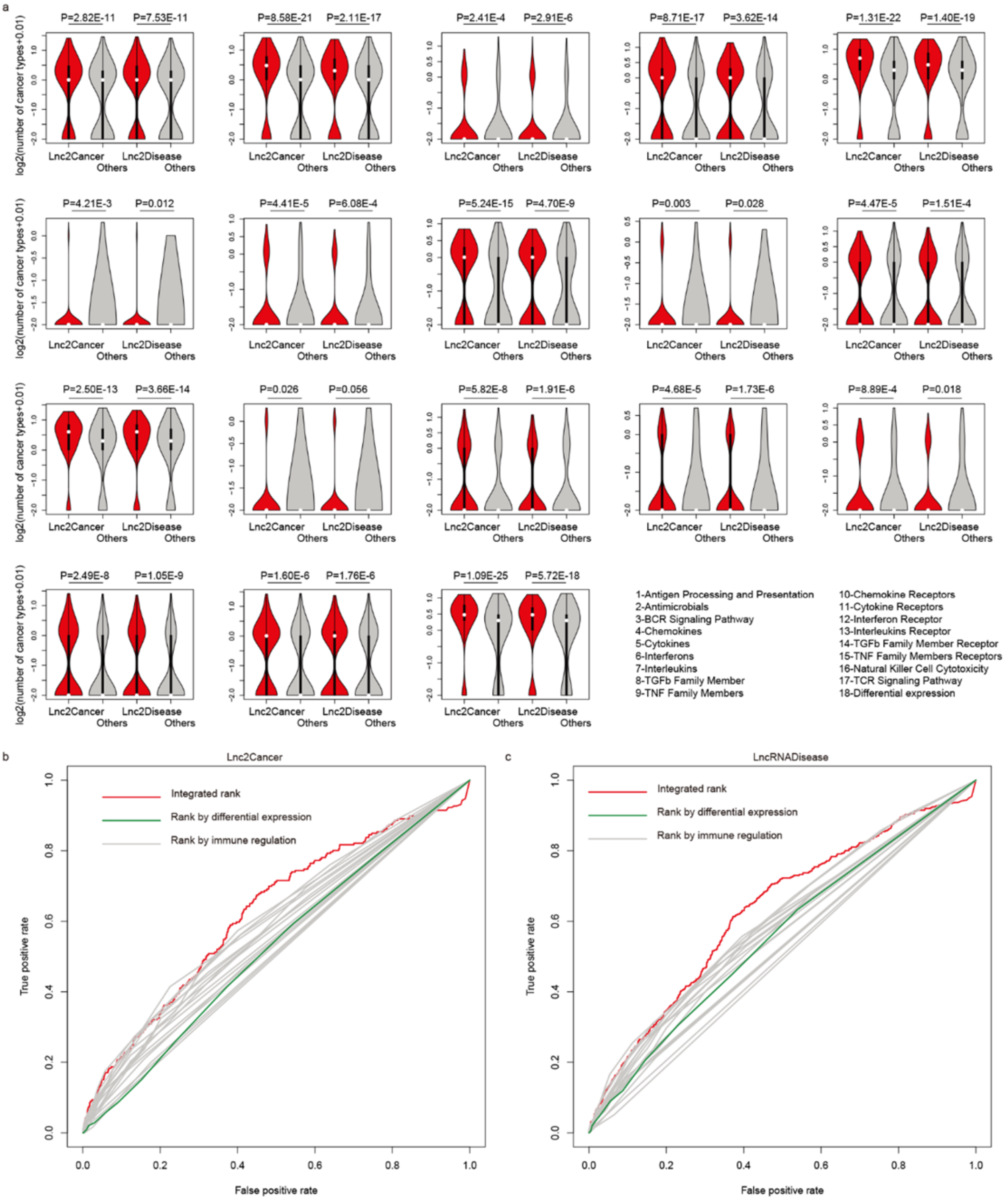

Supplementary Figure 15. ImmLnc helps prioritizing cancer-related lincRNAs. a. Boxplots showing the number of cancer types in which lincRNA–pathway pairs were identified. Red boxes indicate cancer-related lincRNAs and gray boxes indicate other lincRNAs. All p-values are for two-sided Wilcoxon Rank-Sum tests. b. The area under the ROC curve for classification of cancer-related lincRNAs vs other lincRNAs based on Lnc2Cancer data. c. The area under the ROC curve for classification of disease-related lincRNAs vs other lincRNAs based on LncRNADisease data.

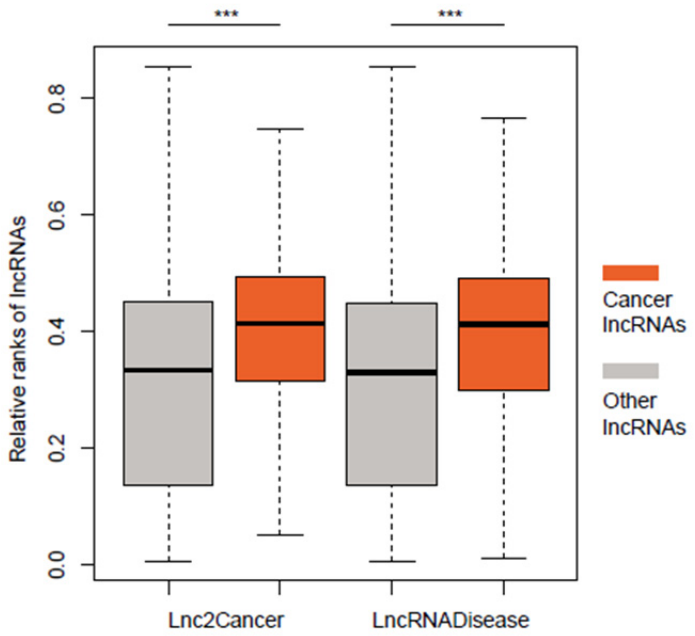

Supplementary Figure 16. The relative ranks of lncRNAs based on the number of cancer types that show an association with immune pathways. The Centre of the boxplots are median values, the bounds of the boxes are 25% and 75% quantiles. The minima are 25% quantile-1.5*interquartile range (IQR) and the maxima are 75% quantile+1.5*IQR. P-values<2.2E-16 for one-sided Wilcoxon Rank-Sum tests.

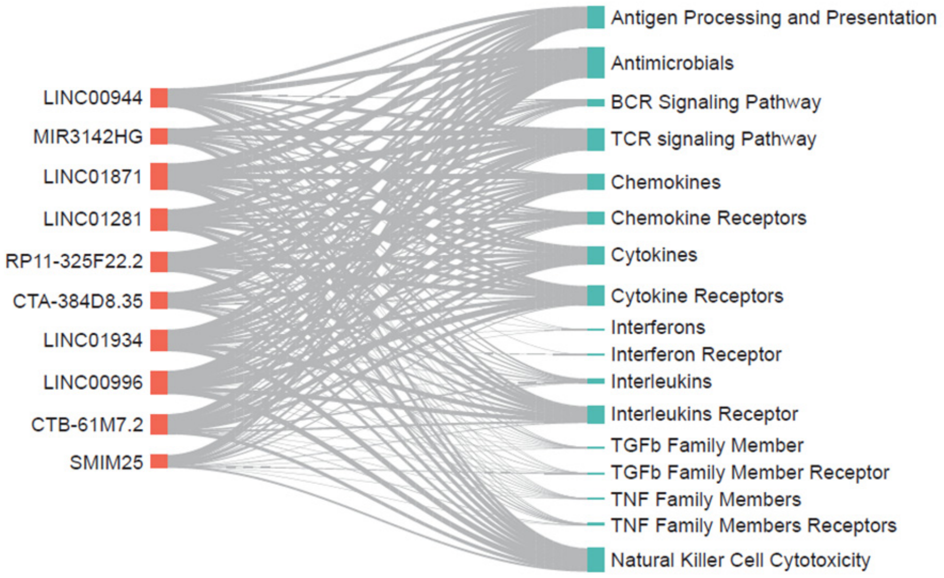

Supplementary Figure 17. River plot showing the association between lncRNAs and immune-related pathways. The weight of the edges corresponds to the number of cancer types showing this association.

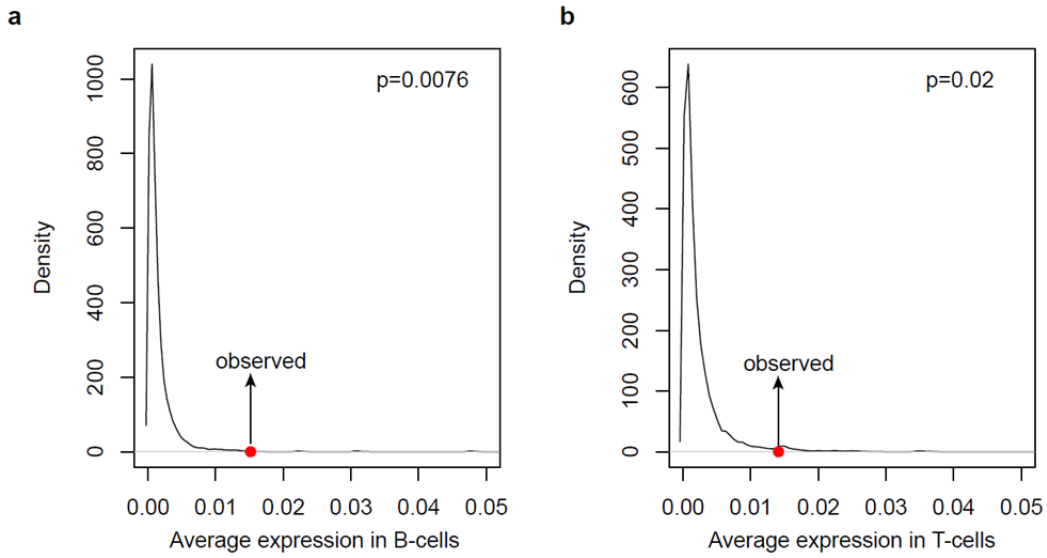

Supplementary Figure 18. The average expression of lncRNA biomarkers was significantly higher than that of randomly selected lncRNAs in B cells and T cells. The lines show the distribution of average expression in random conditions. The red dots represent the observed average expression levels. P-values are for random tests. a, for B cells and b, for T cells.

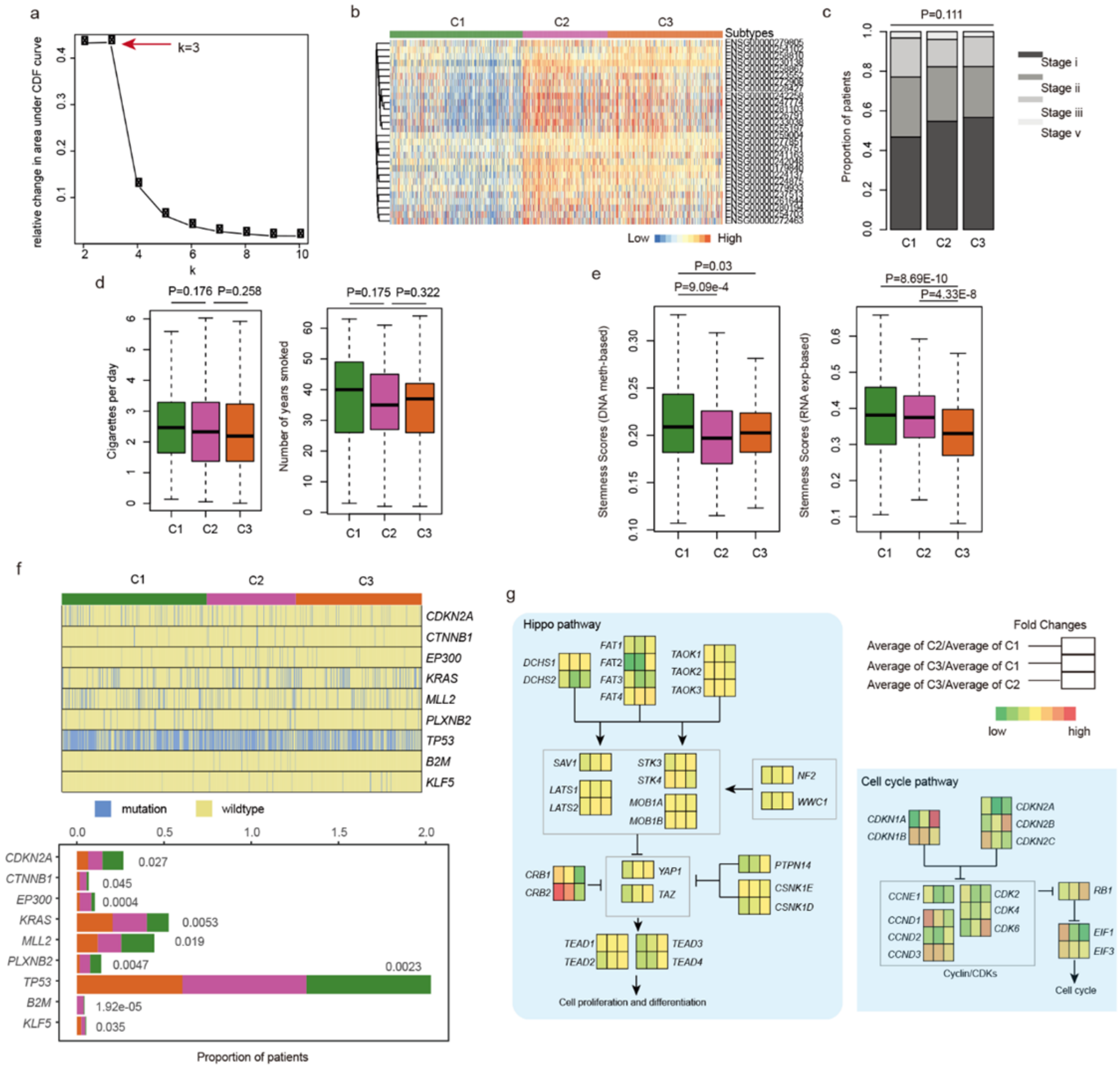

Supplementary Figure 19. The functional characterization of different lung cancer subtypes. a. The relative changes in area under the CDF curve with increasing *k*. b. The expression of 28 lncRNAs in cancer patients of different subtypes. c. The proportion of patients with different stages of the three subtypes. d. The left panel shows the distribution of smoked cigarettes per day for patients of the three subtypes. The right panel shows the distribution of number of years smoked for patients of the three subtypes. P-values for two-sided Wilcoxon Rank-Sum tests. e. The stemness score distribution for patients of different subtypes. The left panel is based on DNA methylation and the right panel is based on RNA expression. P-values for two-sided Wilcoxon Rank-Sum tests. f. The mutation distribution in patients of different subtypes. The top panel shows whether the genes listed on the right are mutated or not in a specific patient. The bottom panel shows the mutation frequency of each gene in different subtypes. g. Diagram of the hippo and cell cycle pathways across the three subtypes. The genes are colored based on the fold change between two subtypes. The Centre of the boxplots are median

values, the bounds of the boxes are 25% and 75% quantiles. The minima are 25% quantile-1.5*interquartile range (IQR) and the maxima are 75% quantile+1.5*IQR.

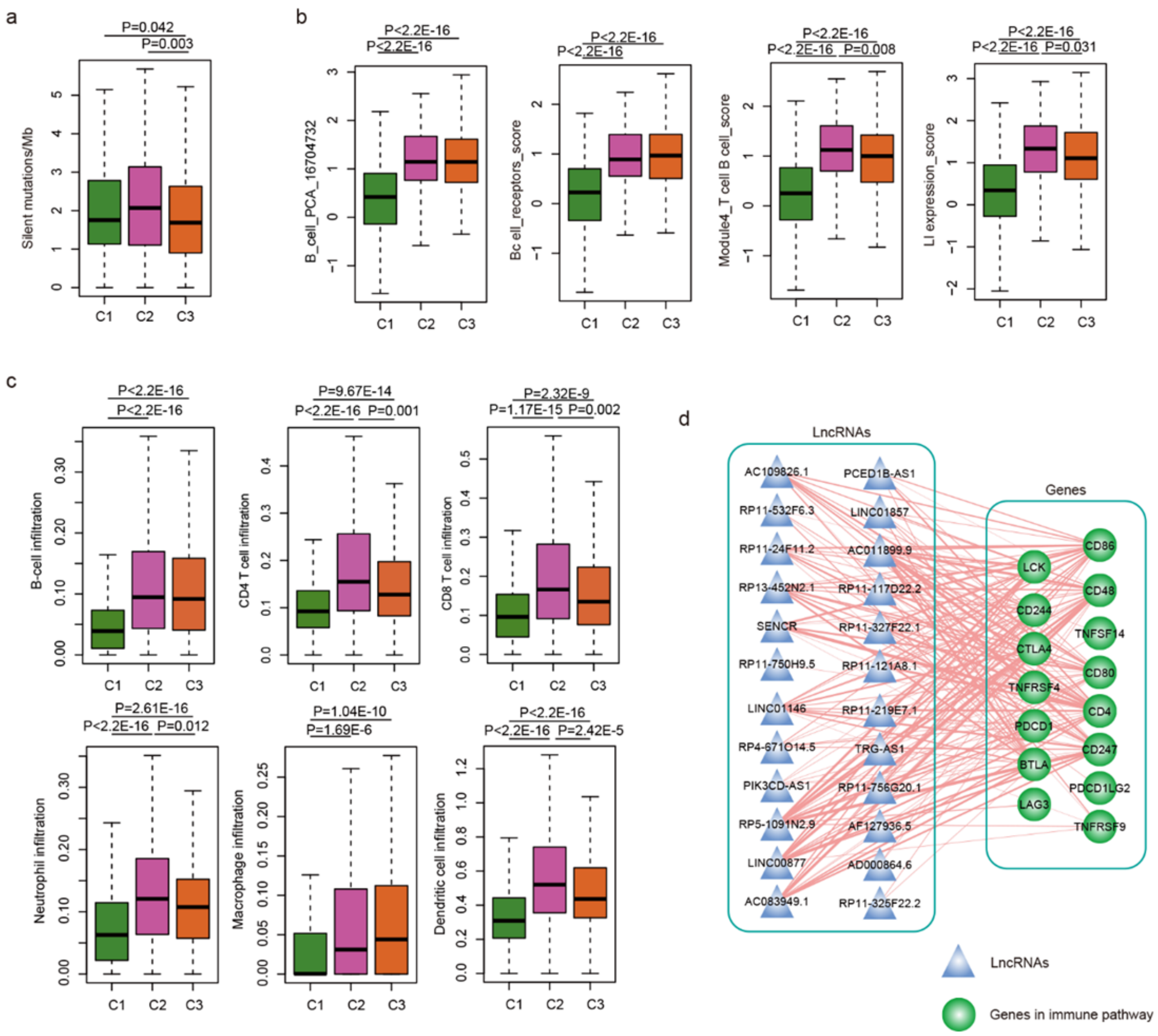

Supplementary Figure 20. The immunology features of patients in different subtypes. a. The number of silent mutations per MB for patients of the three subtypes. P-values for one-sided Wilcoxon Rank-Sum tests. b. The distribution of immune-related gene signature scores across patients of the three subtypes. c. The distribution of immune cell infiltration in patients of different subtypes. P-values for two-sided Wilcoxon Rank-Sum tests in b-c. d. Network showing the correlation between lncRNAs and immune-related genes. The weight of the lines corresponds to the correlation coefficient. Blue indicates lncRNAs and green indicates genes in immune pathways. The Centre of the boxplots are median values, the bounds of the boxes are 25% and 75% quantiles. The minima are 25% quantile-1.5*interquartile range (IQR) and the maxima are 75% quantile+1.5*IQR.

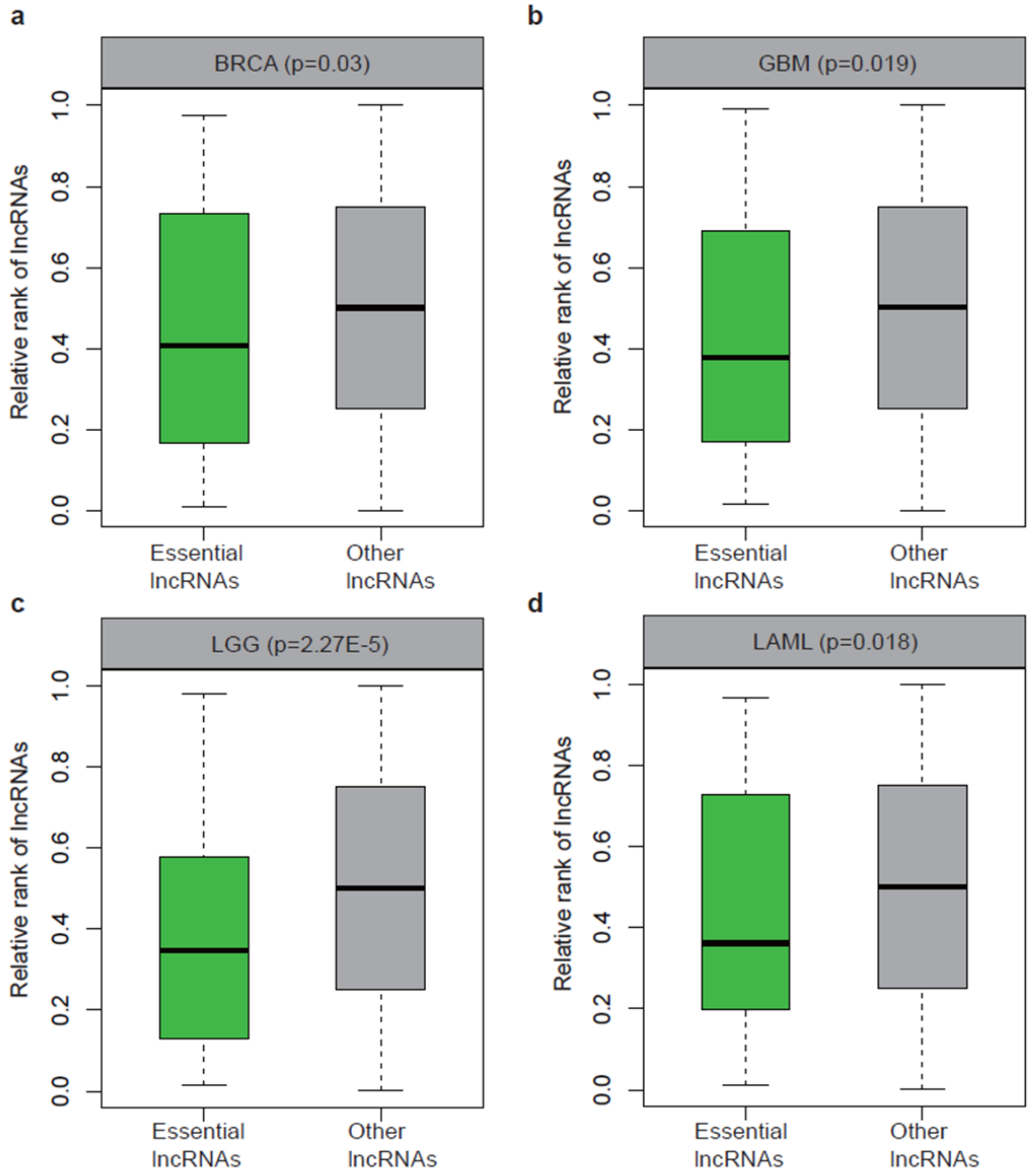

Supplementary Figure 21. Relative rank distribution of essential and other lncRNAs in cancer. LncRNAs were ranked based on their association with cell growth-related gene sets by ImmLnc. The ranks of lncRNAs were normalized and one-sided Wilcoxon Rank-Sum test was used to evaluate the difference between essential lncRNAs and other lncRNAs. The essential lncRNAs were identified by CRISPR-Cas9 screening. a, breast cancer (BRCA); b, glioblastoma multiforme (GBM); c, low grade glioma (LGG); and d, acute myeloid leukemia (LAML). The Centre of the boxplots are median values, the bounds of the boxes are 25% and 75% quantiles. The minima are 25% quantile-1.5*interquartile range (IQR) and the maxima are 75% quantile+1.5*IQR. P-values for two-sided Wilcoxon Rank-Sum tests.

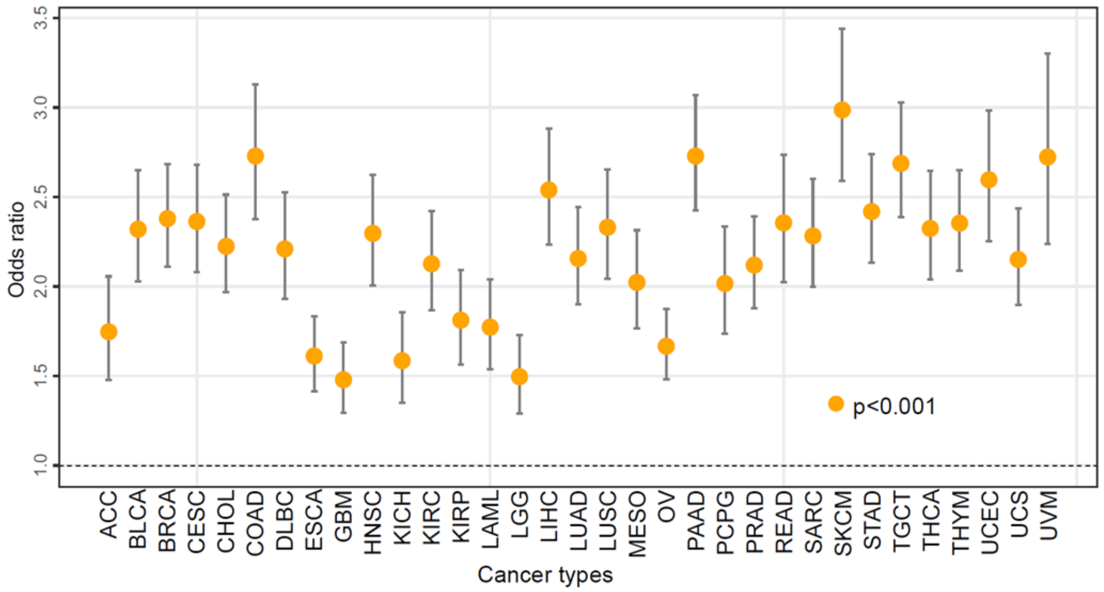

Supplementary Figure 22. The odds ratio distribution in cancer types for comparison of co-occurrence with "immune" in the literature. Two-sided Fisher's exact test was used to test whether the immune-related lncRNAs were more likely to co-occur with "immune" in the literature than other lncRNAs. The error bars were the 95% confidence level of the odds ratio.

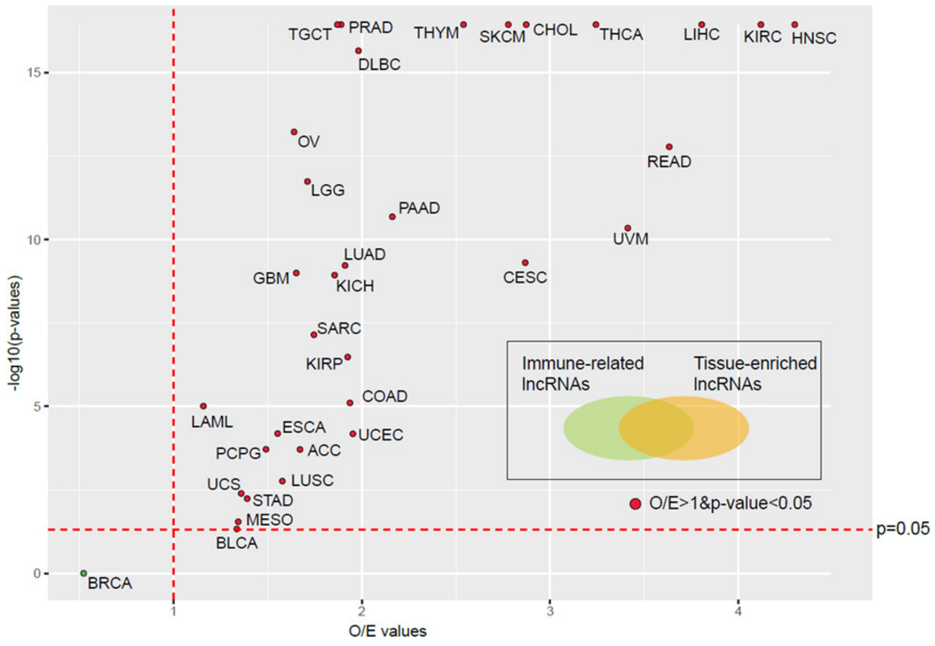

Supplementary Figure 23. Tissue specificity of immune-related lncRNAs. Each dot represents one cancer type; the *x*-axis represents the O/E value and the *y*-axis represents the −log10(*P*-values). Two-sided hypergeometric tests were used.

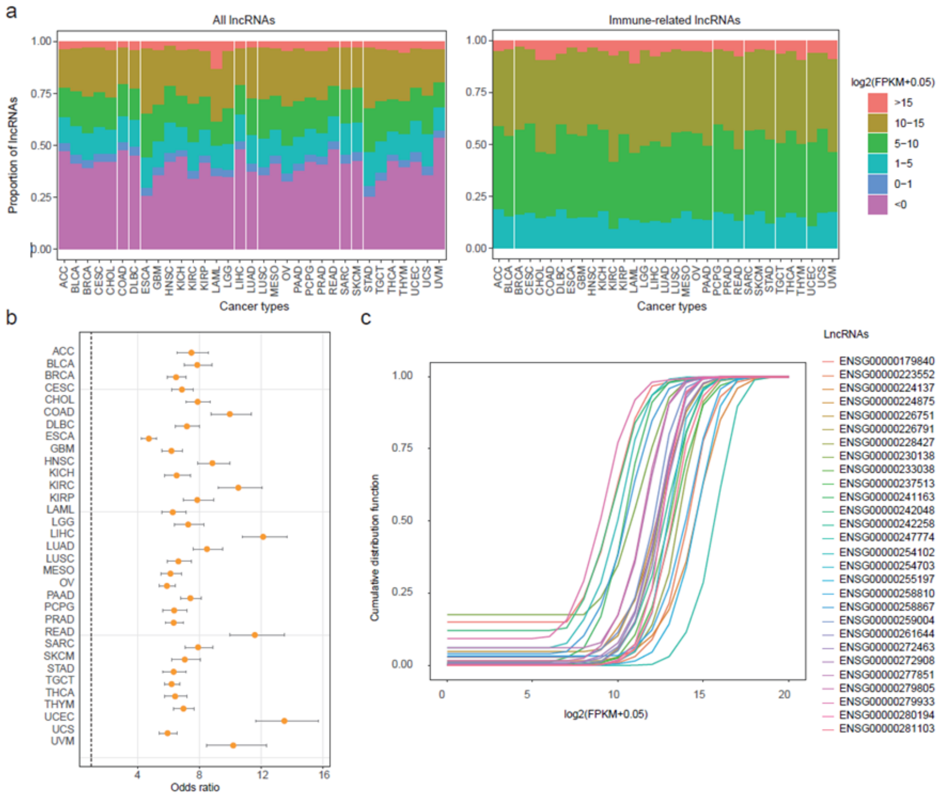

Supplementary Figure 24. Immune-related lncRNAs exhibit high expression in cancer. a. The proportion of lncRNAs with different expression levels in cancer. The left panel shows all lncRNAs, the right panel shows immune-related lncRNAs. B. The odds ratio for two-sided Fisher's exact test. The error bars were the 95% confidence level of the odds ratio. c. The cumulative distribution of the expression of 28 lncRNAs.

References

1. Bao ZS*, et al.* RNA-seq of 272 gliomas revealed a novel, recurrent PTPRZ1-MET fusion transcript in secondary glioblastomas. *Genome Res* **24**, 1765-1773 (2014).

2. Lin X*, et al.* Characterization of Transcriptome Transition Associates Long Noncoding RNAs with Glioma Progression. *Mol Ther Nucleic Acids* **13**, 620-632 (2018).

3. International Cancer Genome C*, et al.* International network of cancer genome projects. *Nature* **464**, 993-998 (2010).

4. Seo JS*, et al.* The transcriptional landscape and mutational profile of lung adenocarcinoma. *Genome Res* **22**, 2109-2119 (2012).

5. Ranzani V*, et al.* The long intergenic noncoding RNA landscape of human lymphocytes highlights the regulation of T cell differentiation by linc-MAF-4. *Nat Immunol* **16**, 318-325 (2015).

6. Schmiedel BJ*, et al.* Impact of Genetic Polymorphisms on Human Immune Cell Gene Expression. *Cell* **175**, 1701-1715 e1716 (2018).

7. Yoshihara K*, et al.* Inferring tumour purity and stromal and immune cell admixture from expression data. *Nature communications* **4**, 2612 (2013).

8. Li Y*, et al.* LncMAP: Pan-cancer atlas of long noncoding RNA-mediated transcriptional network perturbations. *Nucleic Acids Res* **46**, 1113-1123 (2018).

9. Paradis E, Claude J, Strimmer K. APE: Analyses of Phylogenetics and Evolution in R language. *Bioinformatics* **20**, 289-290 (2004).

10. Li T*, et al.* TIMER: A Web Server for Comprehensive Analysis of Tumor-Infiltrating Immune Cells. *Cancer Res* **77**, e108-e110 (2017).

11. Li B*, et al.* Comprehensive analyses of tumor immunity: implications for cancer immunotherapy. *Genome biology* **17**, 174 (2016).

12. Newman AM*, et al.* Robust enumeration of cell subsets from tissue expression profiles. *Nature methods* **12**, 453-457 (2015).

13. Liu SJ*, et al.* CRISPRi-based genome-scale identification of functional long noncoding RNA loci in human cells. *Science* **355**, (2017).

14. Liberzon A, Subramanian A, Pinchback R, Thorvaldsdottir H, Tamayo P, Mesirov JP. Molecular signatures database (MSigDB) 3.0. *Bioinformatics* **27**, 1739-1740 (2011).

15. Ng JCF, Quist J, Grigoriadis A, Malim MH, Fraternali F. Pan-cancer transcriptomic analysis dissects immune and proliferative functions of APOBEC3 cytidine deaminases. *Nucleic Acids Res* **47**, 1178-1194 (2019).

16. Lambrechts D*, et al.* Phenotype molding of stromal cells in the lung tumor microenvironment. *Nat Med* **24**, 1277-1289 (2018).

17. Franzen O, Gan LM, Bjorkegren JLM. PanglaoDB: a web server for exploration of mouse and human single-cell RNA sequencing data. *Database : the journal of biological databases and curation* **2019**, (2019).

18. Xiliang Wang YH, Qiming Zhang, Xianwen Ren, Zemin Zhang. Direct Comparative Analysis of 10X Genomics Chromium and Smart-seq2. (2019).

19. Uhlen M, Hallstrom BM, Lindskog C, Mardinoglu A, Ponten F, Nielsen J. Transcriptomics resources of human tissues and organs. *Mol Syst Biol* **12**, 862 (2016).

20. Uhlen M*, et al.* Proteomics. Tissue-based map of the human proteome. *Science* **347**, 1260419 (2015).

21. Lauss M*, et al.* Mutational and putative neoantigen load predict clinical benefit of adoptive T cell therapy in melanoma. *Nat Commun* **8**, 1738 (2017).

22. Rooney MS, Shukla SA, Wu CJ, Getz G, Hacohen N. Molecular and genetic properties of tumors associated with local immune cytolytic activity. *Cell* **160**, 48-61 (2015).

23. Thorsson V*, et al.* The Immune Landscape of Cancer. *Immunity* **48**, 812-830 e814 (2018).
