# Supplementary material for: Pan-cancer characterization of immune-related lncRNAs identifies potential oncogenic biomarkers
Source: Nat Commun. 2020 Feb 21;11:1000. doi: 10.1038/s41467-020-14802-2 (PMC7035327; doi:10.1038/s41467-020-14802-2)
Supplement: Supplementary file 3 — Description of Additional Supplementary Files [file 41467_2020_14802_MOESM3_ESM.pdf]

Description of Additional Supplementary Files

File Name: Supplementary Data 1
Description: The cancer types used in our analysis.

File Name: Supplementary Data 2
Description: The immune related pathway and genes.

File Name: Supplementary Data 3
Description: The overlap of lncRNA-Pathway association between TCGA data and two other datasets in immune cells.

File Name: Supplementary Data 4
Description: The top ranked 500 lncRNA-pathways across cancer types.

File Name: Supplementary Data 5
Description: The proportion of immune cell infiltration related lncRNAs across cancer types.

File Name: Supplementary Data 6
Description: The p-values for lncRNAs identified in TIMER and CIBERSORT.
